# Development of a SYBR Green Multiplex Real Time PCR for Simultaneous Detection of *Mycobacterium Tuberculosis* and *Nocardia Asteroides* in Respiratory Samples

**DOI:** 10.4314/ejhs.v31i2.6

**Published:** 2021-03

**Authors:** Safar Ali Alizadeh, Amir Javadi, Jalal Mardaneh, Neda Nasirian, Sajjad Alizadeh, Maryam Mohammadbeigi, Siamak Heidarzadeh

**Affiliations:** 1 Medical Microbiology Research Center, Qazvin University of Medical Sciences, Qazvin, Iran; 2 Department of Social Medicine, School of Medicine, Qazvin University of Medical Sciences, Qazvin, Iran; 3 Department of Microbiology, School of Medicine, Infectious Diseases Research Center, Gonabad University of Medical Sciences, Gonabad, Iran; 4 Department of Microbiology, School of Medicine, Student Research Committee, Gonabad University of Medical Sciences, Gonabad, Iran; 5 Department of pathology, School of Medicine, Qazvin University of Medical Sciences, Qazvin, Iran; 6 Medical Doctor, School of Medicine, Tehran University of Medical Sciences, Tehran, Iran; 7 Department of Microbiology and Immunology, School of Medicine, Qazvin University of Medical Sciences, Qazvin, Iran; 8 Department of Microbiology and Virology, School of Medicine, Zanjan University of Medical Sciences, Zanjan, Iran

**Keywords:** Mycobacterium tuberculosis, Nocardia asteroides, Respiratory samples

## Abstract

**Background:**

Nocardia asteroides and Mycobacterium tuberculosis are worldwide-distributed bacteria. These infectious agents can cause many infections in humans, especially in immunocompromised individuals. Pulmonary infections are more common and have similar clinical symptoms. Proper diagnosis and treatment of these patients are important for accurate treatment and could be lifesaving.

**Methods:**

In this study, a multiplex real-time PCR assay was established for the simultaneous detection of the N. asteroides and M. tuberculosis. Both this homemade multiplex real time PCR and routine commercial tuberculosis tests were performed on 150 pulmonary specimens collected from individuals suspected to have tuberculosis.

**Results:**

From 150 specimens, 20 samples were acid fast positive, 14 positives for M. tuberculosis by singleplex real time PCR, 10 positives for N. asteroides by singleplex real time PCR and 2 positives for M. tuberculosis and N. asteroides by multiplex real time PCR whereas 14 samples were positive for M. tuberculosis with commercial test. Differential diagnosis of pulmonary tuberculosis is useful for their proper treatment.

**Conclusion:**

Our test had good performance for differential diagnosis of tuberculosis and nocardiosis. Therefore, it is recommended to be used to diagnose such patients.

## Introduction

*Mycobacterium tuberculosis* (MTB) and *Nocardia asteroides* (NA) are worldwide- distributed bacteria (1,2). These microorganisms can cause many infections in humans especially those who are immunocompromised. The clinical manifestations of these infections are mostly pulmonary (1,3,4). Tuberculosis and nocardiosis often have similar clinical symptoms and radiological view, although extra pulmonary infections have also been reported in the brain, skin and lymphatic systems (1,5,6). In many cases, the bacteria that cause the infection are not properly diagnosed. However, accurate differential diagnosis of the causative agents of infections plays an essential role in their treatment. Usually, the first step in the laboratory diagnosis of these infections is established based on direct tests including gram stain, Ziel-Neelsen, direct fluorescence test and culture methods. Both bacteria are directly seen as gram positive and acid fast positive (7). After direct exams, culture of clinical samples and identification tests are usually performed. Generally, clinical samples are cultured based on the direct study results. The apparent similarity of these bacteria in direct vision can cause the detection process to deviate in the wrong direction. In addition, the cultivation technique for these bacteria, especially MTB is time consuming and prolonged (8,9).

Sputum and bronchoalveolar lavage (BAL) in pulmonary disease, abscesses and skin biopsies in skin infections are typically sent to medical laboratories for diagnosis of in all of these samples, both bacteria can be present as possible causes of infection (10). Prompt and accurate diagnosis of patients will result in timely and appropriate treatment (10,11). Therefore, the development of new methods is necessity for differential diagnosis of nocardiosis and tuberculosis (11).

In order to rapid and efficient Identification of MTB and NA in clinical samples, we developed a multiplex real time PCR that identifies both bacteria at the same time in single tube with high sensitivity and specificity.

## Material and Methods

**Design of primers:** We designed two pairs of dedicated primers to identify MTB and NA using Beacon designer 7 software (Table 1). The melting temperature (Tm) of each primer pair was adjusted so that they did not have a significant distance but the Tm of reaction products has a significant distance to be distinguishable in melting curve analysis. *M. tuberculosis* and *N. asteroides* reference DNA sequences were extracted from Gene Bank (http://www.ncbi.nlm.nih.gov/GenBank). We used the target conserved sequence used in previous studies and encoded the primers (11,12). The primers were evaluated at NCBI database that proved to be unrelated to the genome of other bacteria and human target. The primers were also cross-analyzed for reactivity with together.

**Table 1:** Primer sequences used to identify *Mycobacterium tuberculosis* (MTB) and *Nocardia asteroides* (NA)

Primers names	Primers sequences	Tm of amplicons
*Mycobacterium* *tuberculosis* (MTB)	F: 5′ GACCCGCCAGCCCAGGAT 3′ R: 5′TTCGGACCACCAGCACCTAA 3′	90.2
*Nocardia asteroides* (NA)	F: 5′ TAGGGTGCGAGCGTTGTC 3′ R: 5′CTTCTCAGCGTCAGTTACTTCC 3′	85.9
Beta actin	F: 5′ GTGGGCCGCTCTAGGCACCAA 3′ R: 5′ AAATCGTGCGTGACATCAAAGAG 3′	88.2

**Standard bacterial strains**: *M. tuberculosis* H37R and *N. asteroids* ATCC 19247 were prepared from Pasteur Institute of Iran. The DNA of these bacteria was used to optimize Real Time PCR reactions.

**Optimizing real time PCR reactions:** Singleplex Real time PCR reactions were optimized using each primer pair separately and the standard DNA of each bacterium. These reactions were performed using the Takara SYBR Green master mix and StepOne plus ABI real time PCR equipment. Finally, after optimizing each pair of primers separately, optimization was performed with both primer pairs in single tube. The multiplex real time PCR reaction mixture consisted one microliter (µl) of extracted DNA containing approximately 50 nanogram (ng) of DNA, 10 µl of SYBR Green master mix (2x), 0.4 µl of MTB and NA primers (10 pmol/µl) and deionized water to a final volume of 20 µl. The best physical conditions were optimized to 10 min at 95 degrees as predenaturation, 35 cycles of 95°C for 15 seconds, 60°C for 45 seconds. Finally, the melting curve was drawn from 65°C to 95°C with a gradual increase of 0.11°C. After adjusting the physical and chemical conditions of the multiplex reaction, the melt curve of the reactions was analyzed to confirm the specificity of the reaction. At the end, real time PCR reaction products were analyzed by gel electrophoresis too.

**Evaluation of multiplex real-time PCR**: The sensitivity of optimized multiplex Real time PCR was assayed by preparation serial dilution from 0.5 McFarland bacterial suspensions to 10 bacteria per milliliter. DNA from pellet of each dilution was extracted using the Roche's commercial DNA extraction kit. Specificity was assessed using DNA of non-target bacteria according to other researcher's methods (13). Finally, the reaction repeatability was performed by triplicate. In order to simulate clinical samples, the DNA of target bacteria was mixed with blood in serial dilutions and the ability of the multiplex reaction to detect the target bacteria in the blood was investigated.

**Running multiplex reaction on clinical specimens**: A total of 150 samples were selected from patients referred to the laboratory for pulmonary tuberculosis diagnosis. Sputum and other pulmonary secretions were collected. Initially, all specimens were stained with Ziel Neelsen method and carefully studied by microscopy. DNA of all patient samples were extracted using the Roche's DNA extraction kit after homogenization. The quality of DNA samples was evaluated using Human beta actin gene as control gene. Any samples that had negative response to beta actin gene specific primers were replaced with other ones. Finally, all samples were tested using NA and MTB specific primers singleplex and multiplexed using both pairs of primers. In addition, all samples were tested with a commercial Sinaclone kit for detecting of *M. tuberculosis*. Detection of MTB and NA was done by analyzing the melting curve and Tm of the reaction curve. Tm values were 85.9 for NA and 90.2 for MTB. Because the Tm temperatures of each particular product were sufficiently far apart, NA and MTB were easily distinguishable.

## Results

**Figure 1:**
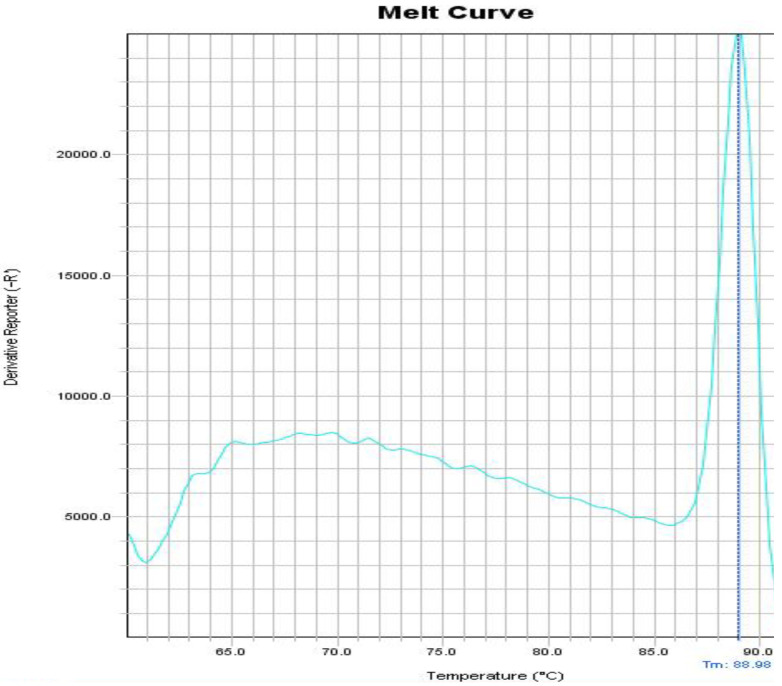
Melting curve of MTB detection alone

**Figure 2:**
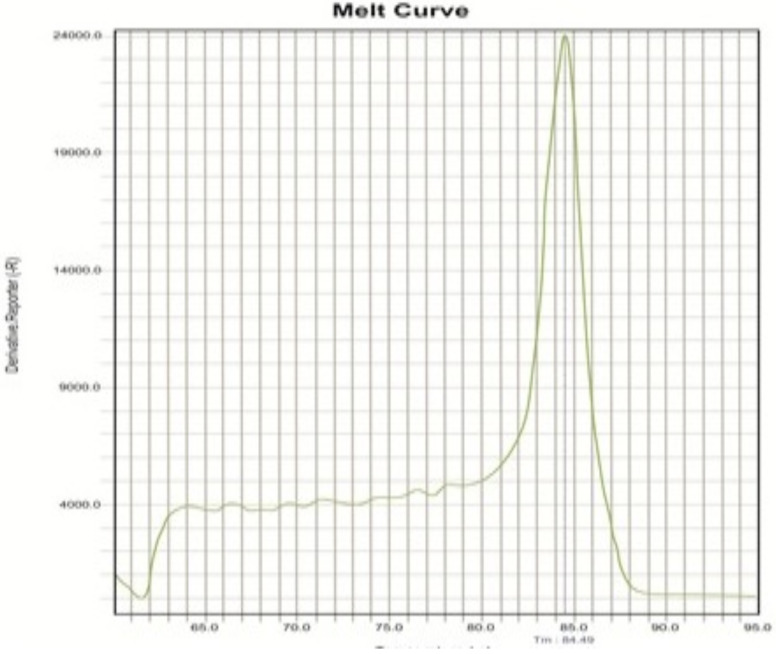
Melting curve reaction of NA detection alone

**Figure 3:**
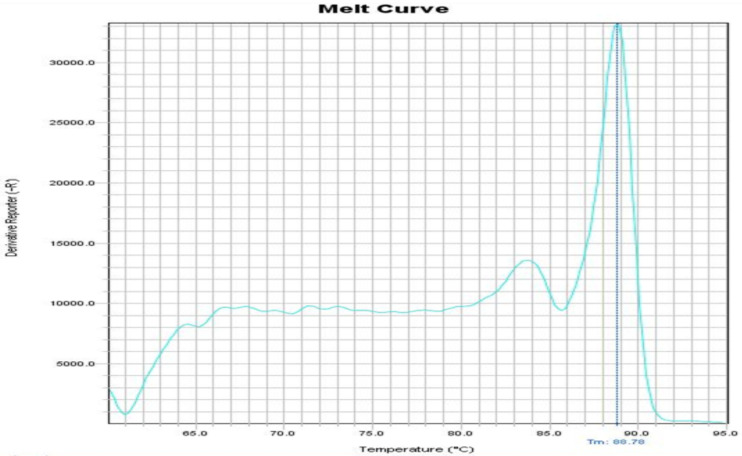
Melting curve PCR reaction of NA and MTB detection in Multiplex Real Time PCR

The NA and MTB genome sequences were downloaded from Gene Bank and the specificity of the two primers was confirmed. In addition, using the NCBI database, it was found that primers do not bind to any target other than their original target such as other bacteria and human, and only match the expected target. Using both primer pairs and standard MTB and NA strains, Real Time PCR reactions were first optimized by singleplex. It was confirmed that the expected bands were being generated. The products of singleplex reaction were sequenced and their accuracy was confirmed. When standard MTB and NA strains were tested by singleplex reactions separately, the Tm for MTB and NA was obtained 90 °C and 85 °C, respectively as our expect. After these steps, the multiplex reaction was optimized using the DNAs of both MTB and NA and the melting curve of the products was exactly similar with the singleplex reaction and nonspecific peak was not observed. A nontemplate reaction was also done as negative control and no amplification plot was observed. Occasionally, due to the formation of a primer dimer in the negative control tube, a non-specific Tm was observed below 75°C (Figures 1, 2 and 3). Intra and Interassay were tested three times and the multiplex reaction repeatability was confirmed. The 0.5-McFarland bacteria suspension was diluted serially. The Real Time PCR was done in the singleplex and multiplex assays for all dilutions. All tests were performed triplicate. The result showed that this method was able to detect 1000 copies, and this ability is stable when the bacteria are mixed with whole blood. As show in Table 2, the multiplex reaction showed 100% concordance with MTB detection kit results. Our reaction is able to detect quickly MTB and NA in clinical specimens by examining the melting curve. The results were in line with the results of the Sina-clone commercial kit. Two samples of patients had a mixture infection MTB and NA, which was well diagnosed with our test.

**Table 2:** Results of homemade multiplex real time PCR and standard tests for *Mycobacterium tuberculosis* (MTB) and *Nocardia asteroides* (NA) detection

Results	Multiplex Real Time PCR	Singleplex Real Time MTB -PCR	Singleplex Real Time NA- PCR	Sina-clone Commercial Kit	Ziel-Neelsen Direct smear
Acid Fast Bacteria	-	-	-	-	20
NA	10	-	10	-	-
MTB	14	14	-	14	-
MTB and NA (mixed)	2	-	-	-	-

## Discussion

Differential diagnosis of infectious agents with similar symptoms is difficult for both physicians and medical laboratories (11). Solving this problem is important for physicians and exciting for microbiology laboratories. Accurate, rapid and easy detection of infectious elements from a variety of clinical specimens has always been the aspiration of microbiologists in laboratories. Sometimes, symptoms of pulmonary nocardiosis are nonspecific and mimic to tuberculosis in clinical feature. The radiological signs of nocardiosis are similar to pulmonary tuberculosis at times. Diagnosis of these infections is controversial (14–16).

Iran has a high potential for developing tuberculosis because of its geographical location and neighboring countries. Iran's proximity to countries such as the Russian Federation with high levels of MDR-Tb and Afghanistan has caused a high prevalence of TB in some geographical areas such as Khorasan, Sistan and Baluchistan (17).

Therefore, the accurate and rapid diagnosis of TB and its differentiation with similar diseases in Iran is highly important. We designed a highly sensitive and precise molecular method capable of detecting both *Mycobacterium tuberculosis* and *Nocardia asteroids* simultaneously in a single test tube. Their validity was verified using NCBI databases. In the laboratory using standard bacteria MTB and NA, each assay was optimized separately and then multiplexed. The primers quality, sensitivity, and specificity were adjusted to excellent level, and analysis of the melting curves of all reactions confirmed that they produced no nonspecific reactions. Finally, this setup test was performed on 150 specimens collected from patients suspected of having tuberculosis. The results showed that this test could be very useful in the differential diagnosis of tuberculosis, nocardiosis and other similar infections.

As shown in Table 2, of the 150 samples of clinically suspected tuberculosis, 20 patients were Ziehl-Neelsen positive. The results of multiplex real time PCR were 10 NA positive and 14 MTB positive. Two patients were positive for NA and MTB simultaneously. Based on these results, it can be concluded that two mix-infected patients were misdiagnosed with routine tests. Other two patients were MTB positive by our test while were negative with Ziel Neelsen method. Therefore, they would be probably misdiagnosed by traditional methods. On the other hand, ten patients who had only nocardiosis, were treated earlier. In addition, for the final diagnosis of patients, it was necessary to wait a month for the results of culture tests.

By careful study of the multiplex test results in our small population of suspected tuberculosis patients, the importance of our test in tuberculosis diagnosis and proper treatment of patients is obvious. However, this test worked well in our study. We suggest that it should be performed in a larger population.
